# Sucralose promotes accumulation of reactive oxygen species (ROS) and adipogenesis in mesenchymal stromal cells

**DOI:** 10.1186/s13287-020-01753-0

**Published:** 2020-06-26

**Authors:** Nabanita Kundu, Cleyton C. Domingues, Jay Patel, Mohammed Aljishi, Neeki Ahmadi, Mona Fakhri, Allison C. Sylvetsky, Sabyasachi Sen

**Affiliations:** 1 The George Washington University, 2300 Eye ST. NW, Washington DC, 20037 USA; 2 Department of Exercise and Nutrition Sciences, Milken Institute School of Public Health, The George Washington University, Washington DC, USA

**Keywords:** Non-nutritive sweeteners, Mesenchymal stromal cells, Adipogenesis, Obesity, Diabetes, Reactive oxygen species

## Abstract

**Abstract:**

Consumption of non-nutritive sweeteners (NNS) has been consistently associated with obesity and cardiometabolic disease in epidemiologic studies. Herein, we investigated effects of sucralose, a widely used NNS, at a cellular level. We wanted to investigate effect of sucralose on reactive oxygen species accumulation and adipogenesis in a human adipocyte tissue-derived mesenchymal stromal cells (MSCs) in a controlled fashion.

**Methods:**

In vitro experiments were conducted on commercially available MSCs obtained from human adipose tissue. hMSCs were exposed with sucralose at 0.2 mM (a concentration which could plausibly be observed in the circulatory system of high NNS consumers) up to 1.0 mM (supra-physiologic concentration) in the presence of both normal and high glucose media to detect a dose response based on the outcome measures. Reactive oxygen species (ROS) were detected using Mitosox Red staining and further analyzed by ImageJ and gene expression analysis. Effect of sucralose on adipogenic differentiation was observed in different concentrations of sucralose followed by gene expression analysis and Oil Red O staining.

**Results:**

Increased ROS accumulation was observed within 72 h of exposure. Increased adipogenesis was also noted when exposed to higher dose of sucralose.

**Conclusion:**

Sucralose promotes ROS accumulation and adipogenesis in human adipose tissue derived mesenchymal stromal cells.

## Introduction

Consumption of added sugars is associated with the development of obesity, diabetes, high blood pressure, cardiovascular disease (CVD), and dyslipidemia [1–4]. The American Heart Association (AHA) recommends that calories from added sugar should not exceed 100 or 150 kcals per day for adult females and males, respectively [5]. Non-nutritive sweeteners (NNS) are commonly used as a replacement for added sugar, as they are sweet but contain no or few calories. The US Food and Drug Administration (FDA) regulates six NNS (saccharin, acesulfame-potassium, sucralose, aspartame, neotame, and advantame) and sets acceptable daily intake limits for each individual sweetener [6].

The *National Health and Nutrition Examination Survey* (*NHANES*) 2009–2012 [7] reported 25% of children and more than 41% of adults consume NNS directly or indirectly in foods and beverages in the USA. Whether as NNS have beneficial effects on cardiometabolic health or rather promote weight gain and metabolic dysfunction in humans remains controversial. Numerous epidemiologic studies have shown associations between NNS consumption with obesity, diabetes, and stroke [8–11]. Potential mechanisms for these observations have been identified in in vitro and in vivo studies. For example, in human functional magnetic resonance imaging studies, less reward (activation of the dopaminergic system) was observed when subjects ingested NNS compared to glucose [12]. In mice, saccharin exposure led to changes in the gut microbiome causing glucose intolerance [13]. Similarly, aspartame also caused microbiome changes and altered short-chain fatty acid production [14]. In mature mouse adipocytes, NNS exposure promoted adipogenesis and suppressed lipolysis [15]. In this study, we focused on the effects of sucralose, one of the widely used NNS, on ROS accumulation and adipogenesis of human subcutaneous adipose tissue-derived mesenchymal stromal cells (hMSCs).

## Methods

### In vitro cell culture

#### ROS production detection in presence of normal glucose and high glucose and corresponding gene expression profile

Human mesenchymal stromal cells (hMSCs) were obtained from Lonza Inc.(Walkersville, MD, USA). They were cultured in Dulbecco’s modified Eagle’s medium (DMEM). hMSCs were exposed to high (25 Mm) and normal (5.5 Mm) glucose media in the presence of sucralose (0 mM, 0.2 mM, 0.45 mM, and 1 mM) for 72 h followed by gene expression analysis and Mitosox Red staining. The gene expression profile was directed towards adipogenesis (CEBPa, PPARG), antioxidants (SOD 1, SOD2, SOD3, Catalase, GPX1, GPX3), and glucose transporters (GLUT1 and GLUT4). We choose 72 h as the time point for all our experiments to observe discernable intracellular ROS accumulation with Mitosox staining.

#### Detection of adipogenesis in presence of adipogenic media

hMSCs were exposed to adipogenic media (Lonza, Walkersville, MD, USA) in the presence of sucralose (0 mM, 0.2 mM, and 1 mM) for 3 cycles of induction and 3 cycles of maintenance (18 days) followed by gene expression analysis and Oil Red O staining.

#### RNA extraction, cDNA synthesis, and gene expression in hMSCs and subcutaneous fat

Quantitative reverse transcriptase polymerase chain reaction (qRT-PCR) was used for gene expression analysis. Total mRNA from hMSCs was isolated by RNeasy Mini Kit (Qiagen, Hilden, Germany). T100 Thermal Cycler (Bio-Rad, Hercules, CA) was used to convert mRNA to cDNA using the High Capacity cDNA Reverse Transcription Kit (Applied Biosystems, Foster City, CA). CFX96 Real-Time qPCR System (Bio-Rad) was used to analyze the genes of interest using TaqMan Universal Master Mix II (Applied Biosystems, Foster City, CA). Expression of individual genes was normalized to housekeeping genes (18S or GAPDH).

### Mitosox Red staining

hMSCs were exposed to normal glucose (5.5 mM) and high glucose (25 mM) with 0 mM, 0.2 mM, 0.45 mM, and 1.0 mM sucralose for 72 h. For Mitosox Red staining, 5 μM working solution was made from the stock solution (5 Mm) by adjusting with HBSS/Ca/Mg buffer. Cells were incubated in 1 ml of 5 μM Mitosox Red for 20 min at 37 °C followed by washing with Hanks’ Balanced Salt Solution (HBSS/Ca/Mg) buffer and fixed with 4% paraformaldehyde. Intensity of fluorescence was estimated by ImageJ.

### Oil Red O staining

To prepare the stock solution of Oil Red O (Sigma), 0.5% Oil Red O was dissolved in isopropanol. Three parts of this stock solution were mixed with 2 parts of phosphate-buffered saline (PBS) to make working Oil Red O solution. hMSCs were stained by Oil Red O working solution for 20 min, followed by 3 washes with PBS.

### Cell viability test

Cell viability was performed using trypan blue exclusion test, during the same setting as the differentiation experiment.

### Statistical analysis

Results were analyzed by using unpaired *t* test with *p* values *< 0.05 considered statistically significant.

## Results

### Mitosox Red staining of cells exposed to normal and high glucose with or without sucralose

**Fig. 1:**
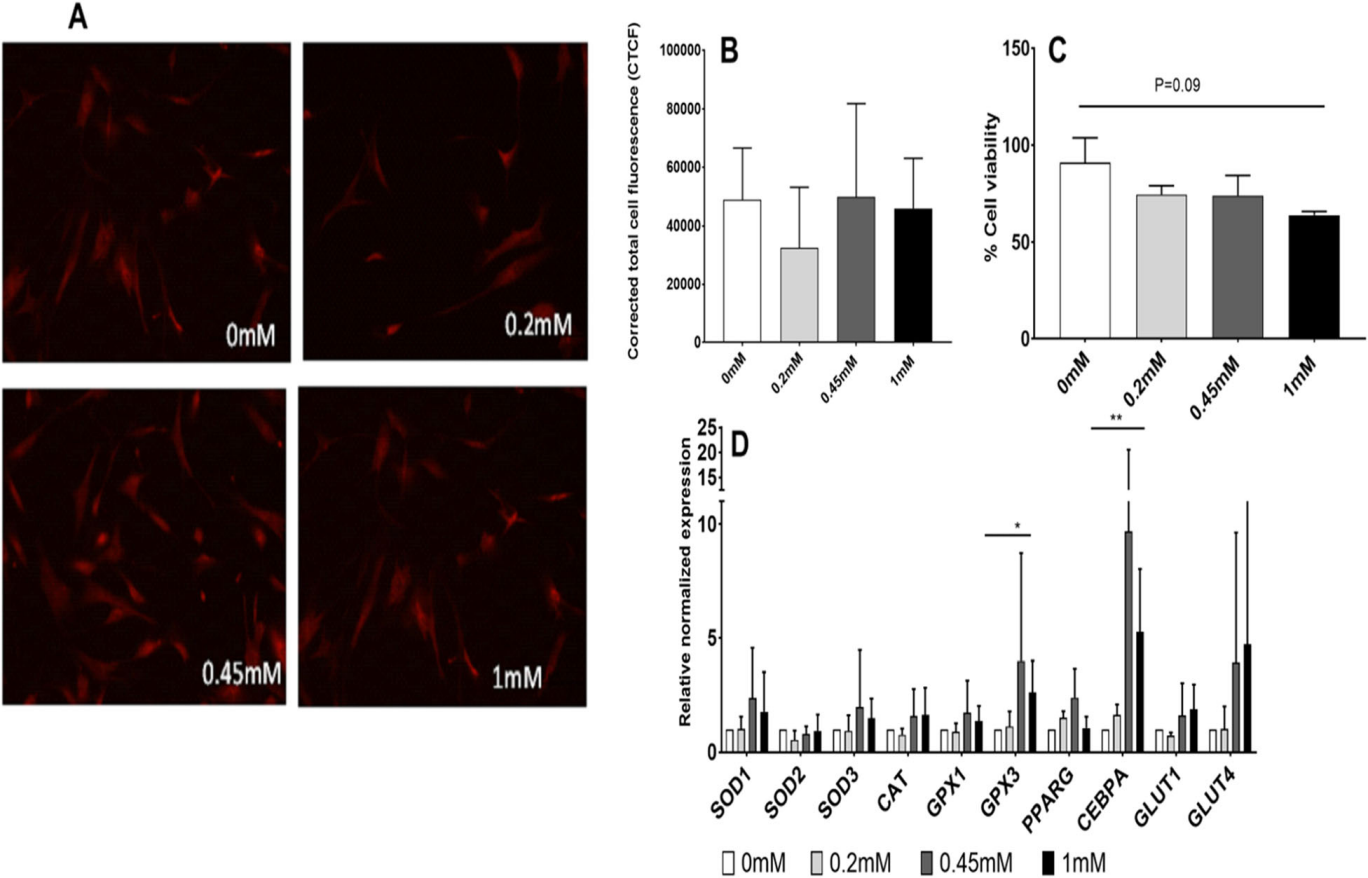
Experiments in normal glucose DMEM (5.5 mM). **a**, **b** Effect of sucralose on reactive oxygen species (ROS) accumulation has been tested in vitro by Mitosox staining (*n* = 3). Accumulation of ROS in human mesenchymal stromal cells (MSCs) increased secondary to sucralose exposure in a dose-dependent manner. **a** shows florescence with Mitosox Red in presence of different concentrations of sucralose by confocal microscopy. **b** shows relative florescence measured from MSCs exposed to different concentrations of sucralose. **c** Shows MSC viability following exposure to sucralose. Viability of cells (*N* = 2) decreased, when exposed to sucralose in a dose-dependent manner in the presence of normal glucose concentrations in culture medium. **d** Effects on antioxidants and adipogenic genes in a dose-dependent manner when MSCs were exposed to sucralose in the presence of normal glucose. Statistical analysis: unpaired *t* test was performed by considering NG (normal glucose) as control (*N* = 2)

The presence of reactive oxygen species (ROS) was examined by Mitosox Red staining in the presence of normal glucose: no statistically significant changes in ROS accumulation were observed in any of the sucralose-exposed conditions (0.2 mM, 0.45 mM, 1 mM) (Fig. 1a, b).

A trend of reduction (1.4-fold, *p* = 0.09, between 0 and 1 mM) in MSC viability was observed with increasing doses of sucralose (Fig. 1c). A major reduction in cell viability was noted between 0 and 0.2 mM.

Significant upregulation of antioxidant gene such as extra-cellular glutathione peroxidase (GPX3) and gene associated with adipogenic differentiation such as CCAAT/enhancer-binding protein alpha (CEPBa) genes was observed in response to sucralose exposure with the same set of cells mentioned above in Fig. 1a. Here, MScs were exposed to normal glucose media containing sucralose concentrations of 0.2 to 1 mM.

When human mesenchymal stromal cells (hMSCs) were exposed to 0 mM and 1 mM sucralose (Fig. 1d), the main upregulated genes again were GPX3 and CEBPA (2.6- and 5.2-fold, *p* = 0.03 and 0.008, respectively). The gene upregulation was not noted at 0.2 mM but noted at 0.45 mM and 1 mM sucralose concentrations.

**Fig. 2:**
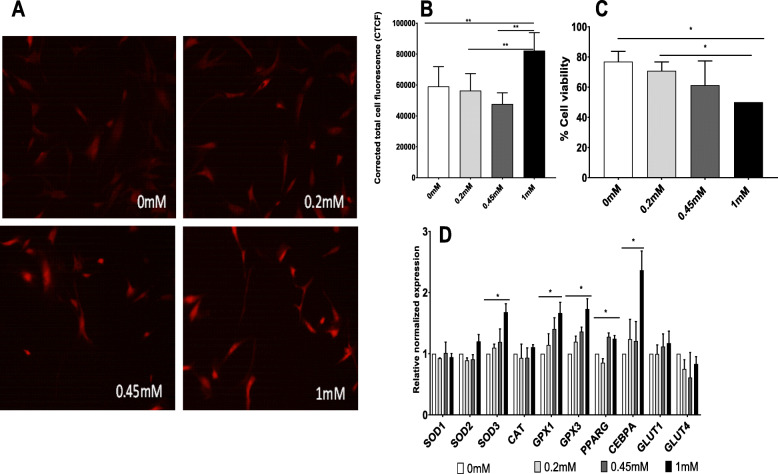
Experiments in the presence of high glucose DMEM (25 mM). **a**, **b** Effect of sucralose on reactive oxygen species (ROS) accumulation has been tested in vitro by Mitosox staining (*n* = 3). Accumulation of ROS in human mesenchymal stromal cells (MSCs) increased secondary to sucralose exposure in a dose-dependent manner. Response of ROS accumulation in the presence of sucralose appears to be accentuated in the presence of higher levels of glucose in the cell media. **c** Viability of cells (*N* = 2) when exposed to sucralose in a dose-dependent manner in the presence of high glucose showed decreasing cell viability in increasing concentrations. **d** Effects on antioxidants and adipogenic genes in a dose-dependent manner when MSCs were exposed to sucralose in the presence of high glucose. Statistical analysis: unpaired *t* test was performed by considering HG (high glucose) as control (*N* = 2 for qPCR)

When the presence of reactive oxygen species (ROS) was examined in the presence of high glucose (DMEM 25 mM glucose) using Mitosox Red staining, elevated ROS accumulation was observed (Fig. 2a, b). ROS accumulation increased (1.4-, 1.4-, and 1.7-folds, *p* = 0.009, 0.001, and 0.0001, respectively) when cells were exposed to 1 mM sucralose in comparison to control (absence of sucralose) (by ImageJ analysis,).

Of note, cell florescence secondary to ROS accumulation was higher (above 60,000 CTFC units) in all conditions in the presence of high glucose, compared to normal glucose.

Cell viability analyses (by trypan blue exclusion method) showed a decrease of 1.5-fold and 1.4-fold respectively (*p* = 0.03 and 0.03, respectively) when cells were exposed to 1 mM sucralose in comparison to 0 mM sucralose and 0.2 mM (Fig. 2c). There was a decrease in viability between 0.2 and 1 mM indicating accumulating effect of cell toxicity in a dose-dependent fashion.

We also observed significant upregulation of antioxidant and adipogenic differentiation genes with same sets of cells mentioned previously in Fig. 2a when cells were exposed to high glucose (Fig. 2d). Genes including SOD3 (superoxide dismutase 3, an extra-cellular antioxidant), GPX1 (glutathione peroxidase 1, an cytosolic antioxidant), and GPX3 (1.6-, 1.6-, and 1.7-fold, *p* = 0.01, 0.03, and 0.02, respectively) were also upregulated in this experimental set when comparing human mesenchymal stromal cells (hMSCs) exposed to 0 mM and 1 mM sucralose.

Adipogenic genes such as CEBPa and PPARG were upregulated in an increasing dose-dependent fashion of sucralose concentration, in the presence of high glucose (HG).

Viability of hMSC was decreased in the presence of high glucose in comparison to normal glucose (Figs. 1c and 2c) in the same concentration of sucralose.

### Effects of sucralose on adipogenic differentiation

**Fig. 3:**
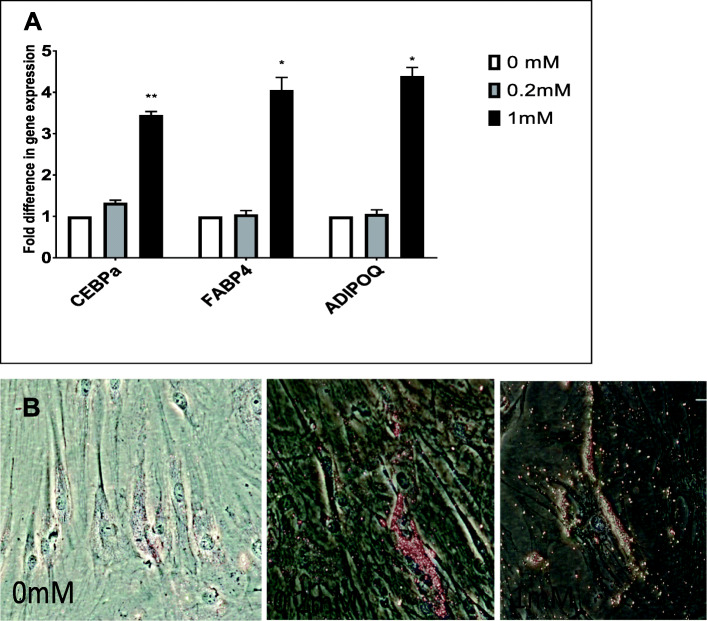
**a** Effects of sucralose on human mesenchymal stromal cells (MSCs) at 18 days observed by gene expression analysis. Genes associated with increased intracellular fat, such as CEBPA, FABP4, and adiponectin, upregulated in a dose-dependent manner. Clearly high expression levels were noted at 1.0 mM, but there was clear upregulation of adipogenic genes in the presence of 0.2 mM (a physiological dose). Statistical analysis was performed by considering 0 mM as control (*n* = 2). The mRNA expressions were compared to relative values to adipogenic media without sucralose (0 mM). **b** Effect of sucralose on adipogenic differentiation. MSCs were exposed to adipogenic media for 18 days followed by Oil Red O staining. Increasing number of oil droplet with higher concentrations of sucralose indicates the increased presence of intracellular fat. Cells at 1.0 mM sucralose are sparse, but viable cells were packed with lipid droplets

hMSCs were exposed to adipogenic media (Lonza, Walkersville, MD, USA), to mimic an obesogenic environment. Zero millimolar, 0.2 mM, and 1 mM sucralose were added to the adipogenic media and cultured for 18 days (much longer period than 72 h, following standard differentiation times for MSCs). Gene expression analysis demonstrated a relative upregulation of genes associated with increased intracellular fat, such as CEBPA, FABP4 (fatty acid binding protein or adipocyte protein 2 is a carrier protein for fatty acid and primarily expressed in adipocytes), and ADIPOQ (adiponectin, a protein hormone produced in mature adipocytes), 2.05-fold, 3.45-fold, and 3.5-fold with *p* values less than 0.05, respectively (Fig. 3a).

We also stained hMSCs exposed to adipogenic media and sucralose with Oil Red O to identify oil droplet (Fig. 3b), as an indication of the adipogenesis process.

## Discussion

Our in vitro data showed increased adipogenesis and antioxidant expression with sucralose in a dose-dependent manner in a high glucose environment. There appears to be a compensatory upregulation of antioxidant genes, in response to increased intracellular ROS, noted on Mitosox Red staining [16–18]. We detected quantifiable amount of Mitosox Red (Thermo-Fisher) staining at 72 h, though minimal staining was evident as early as 24 h. Notably, ROS accumulation is also reported in response to increased sugar intake and promotes the development of cardiovascular disease [19]. Our data confirms that there is increased ROS accumulation (by florescence strength) in the presence of high glucose compared to normal glucose [20].

It is important to distinguish cellular ROS or mitochondrial ROS presence difference. The main sources of cellular ROS are mitochondria and NADPH oxidases (NOXs). ROS produced in the mitochondria (mtROS) compared to NOX-generated ROS were initially considered to be unwanted by-products of oxidative metabolism though recent evidence indicates that mtROS have been incorporated into signaling pathways including those regulating immune responses, inflammation, autophagy, and cell differentiation. Mitosox Red stain from Thermo-Fisher specifically targets the mitochondria and is rapidly oxidized by superoxide which is primarily produced in the mitochondria. In our figures, we see Mitosox Red staining in the cytosol; however, we believe that the source of ROS is the mitochondria.

While our in vitro findings require further corroborative studies in vivo, our results suggest that consuming sucralose may promote metabolic dysfunction by promoting ROS accumulation intracellularly which initially starts in the mitochondria, and subsequently, the ROS and Mitosox staining is evident in the cytosol. We have previously shown that ROS accumulation (in conditions such as hyperglycemia) can be associated with increased adipogenic differentiation of human MSCs. We noted increased ROS accumulation in the presence of high concentrations of sucralose in normal and high glucose media with concomitant increase in adipogenic genes such as CEBP-alpha. The increased intracellular ROS accumulation appears to be more pronounced in the presence of high glucose compared to normal glucose. Therefore, these findings may be particularly relevant to a hyperglycemic milieu in a clinical setting such as diabetes compared to a state without diabetes. More importantly, the ROS accumulation features were noted quite definitively, within 72 h of exposure to sucralose either in normal or in high glucose, indicating the relative quick onset of ROS production and accumulation following sucralose exposure.

Our previous work on human adipogenic MSCs have shown mitochondrial complex 1 dysfunction in the presence of high glucose secondary to superoxide activity. We demonstrated that upregulation of a mitrochondrial antioxidant such as superoxide dismutase 2 (SOD2) reduced oxidative stress and prevents adipogenesis. Similarly, increased intracellular ROS in the presence of sucralose is expected to impair mitochondrial function with subsequent less energy production and accumulation of substrate such as glucose accumulation as lipid droplets (as seen in this case) leading to increased adipogenesis [20, 21].

The increased intracellular ROS accumulation, post-sucralose exposure, most likely triggers reactive antioxidant gene mRNA upregulation, which could be an acute response reaction to increased intracellular ROS accumulation.

It may be worthwhile to do follow-up studies to investigate the duration of this response in relation to ROS production secondary to sucralose exposure. However, the degree of ROS production and duration of action would depend on plasma sucralose levels.

As sucralose may manipulate glucose transporter expression, we tested two glucose transporters’ (GLUT1 and GLUT4) mRNA expression in the presence of normal and high glucose with varying concentrations of sucralose; we did not find any obvious differences in expression across the concentrations (Figs. 1d and 2d) indicating that GLUTs may not play a role in sucralose effects on human MSCs.

Lastly, our results indicate increased mature fat droplet accumulation in vitro, as seen under light microscope in the presence of higher concentration of sucralose, at both 0.2 and 1 mM, at day 18, of adipogenic media exposure. As mentioned before, 0.2 mM sucralose in culture media will be closer to physiological level that can be achieved on NNS consumption [7, 13].

It may be speculated that early production of ROS, by 72 h, leads to increased mature fat-like cells (with increased fat droplets) by day 18, associated with mature fat genes such as FABP4 and ADIPOQ along with fat transcription factor gene upregulation such as CEBP-alpha.

## Conclusion

The experiments that we have described here are a prelude to human in vivo mechanistic studies. There are several human studies that epidemiologically indicate a possible connection between artificial sweetener consumption and metabolic dysregulation. Our experiments are novel and for the first time establish a possible cell-based mechanism of how sweeteners may influence differentiation of fat-based stem cells in humans and promote adipogenesis. Based on our literature search, this is the first study to use human adipose tissue-derived hMSCs to discern the effect of sucralose on fat precursors in humans. These cells are multipotent cells and as per embryology can differentiate into myocytes, chrondrocytes, osteocytes, and adipocytes depending on the cellular milieu [20]. Certain conditions augment their differentiation towards adipogenesis, and increased intracellular ROS accumulation is one of those factors [20, 21]. Artificial sweetener such as sucralose promotes intracellular ROS accumulation in a dose-dependent fashion and more so in a hyperglycemic milieu compared to normoglycemic milieu at all concentrations of sucralose. The interesting part is that the ROS production occurs almost immediately and discernable by 72 h in spite of no obvious cell toxicity.

Our results highlight the need for further in vivo studies, measuring production and accumulation of reactive oxygen species, in a dose-dependent fashion as well as increased fat accumulation, leading to increased insulin resistance and CVD. In fact, our in vivo study [22] indicates that sucralose promotes acute inflammatory response and our in vitro results suggest that rapid and immediate ROS accumulation may be the mechanism. Our results explain adverse associations between NNS consumption and cardiometabolic health reported in epidemiological studies at a cellular level.

## Data Availability

All materials are freely available on written request to Dr. Sen.
